# Kinematic alignment preserves the mid‐flexion trochlear line orientation in total knee arthroplasty: A prospective analysis from the FP‐UCBM Knee Study Group

**DOI:** 10.1002/ksa.12810

**Published:** 2025-08-19

**Authors:** Stefano Campi, Giancarlo Giurazza, Edoardo Franceschetti, Marco Edoardo Cardinale, Andrea Tanzilli, Pietro Gregori, Michele Paciotti, Biagio Zampogna, Umile Giuseppe Longo, Rocco Papalia

**Affiliations:** ^1^ Fondazione Policlinico Universitario Campus Bio‐Medico Roma Italy; ^2^ Department of Medicine and Surgery, Research Unit of Orthopaedic and Trauma Surgery Università Campus Bio‐Medico di Roma Roma Italy

**Keywords:** kinematic alignment (KA), mid‐flexion trochlear line (MTL), total knee Arthroplasty (TKA), trochlear restoration

## Abstract

**Purpose:**

Kinematic alignment (KA) in total knee arthroplasty (TKA) aims to restore the patient's native joint anatomy by resurfacing the distal and posterior femoral condyles. However, the trochlear anatomy is often overlooked, raising concerns about potential relative internal rotation of the femoral component. The aim of this study was to define the 𠄈mid‐flexion trochlear line’ (MTL) and assess its orientation relative to the posterior condylar line, hypothesising a parallelism between the two.

**Methods:**

A total of 158 knees (145 patients) undergoing KA TKA were prospectively analysed, after excluding post‐traumatic osteoarthritis, cases with trochlear dysplasia, and femoral component flexion >5°. The anterior chamfer cut was conducted with a posterior referencing guide and the most prominent points of the medial and lateral trochlear facets—defining the MTL—were measured with a caliper. The MTL orientation relative to the posterior condylar line was calculated as the difference between the medial and lateral trochlear facets, with the two lines considered parallel for differences of 0 ± 1 mm. Two one‐sided tests was implemented to assess equivalence between the two lines within a ±1 mm threshold. Correlations with coronal plane parameters (hip‐knee‐ankle angle [HKA], medial proximal tibial angle [MPTA] and lateral distal femoral angle [LDFA]) were assessed with Pearson's correlation coefficient. Statistical significance was set at *p* < 0.05.

**Results:**

The mean difference between the medial and lateral trochlear facets was 0.1 ± 1.40 mm, with 81.7% of cases falling within the 0 ± 1 mm range, indicating parallelism between the posterior condylar line and the mid‐flexion trochlear line (*p*  =  0.709). No significant correlations were observed between MTL orientation and HKA, MPTA or LDFA.

**Conclusions:**

Referencing the posterior condylar line accurately restores MTL orientation in the vast majority of patients, irrespective of coronal plane parameters. These findings support the biomechanical rationale of kinematic alignment, dispelling concerns about femoral component internal rotation.

**Level of Evidence:**

Level IV, prospective observational study.

**Abbreviations**
FA: functional alignment
HKA: hip‐knee‐ankle angle
ICC: intra‐class correlation coefficient
KA: kinematic alignment
LDFA: lateral distal femoral angle
MA: mechanical alignment
MPTA: medial proximal tibial angle
MTL: mid‐flexion trochlear line
PCA: posterior condylar axis
SD: standard deviation
TKA: total knee arthroplasty
TOST: two one‐sided tests

## INTRODUCTION

Kinematic alignment (KA) is a pure resurfacing technique, aiming to restore the patient's specific pre‐arthritic joint anatomy. However, KA's current emphasis is directed to restoring distal and posterior femoral condylar surfaces [3], without specifically addressing the native anatomy of the trochlea. Specifically, one of the main criticisms of KA is its potential to induce relative internal rotation of the femoral component. In fact, in contrast to mechanical alignment (MA)—where the femoral component is typically implanted with 3° of external rotation relative to the posterior condylar line—and functional alignment (FA)—where femoral rotation is adjusted to balance the flexion gap—KA keeps the femoral component rotation parallel to the posterior condylar line. This concept is consistent with one of the key principles of KA, which is the parallelism between the posterior condylar axis (PCA)—around which the tibia flexes and extends—and the “patellar transverse axis”, around which the patella flexes and extends [3, 4, 10, 19].

While recent literature increasingly emphasises the importance of restoring the anatomy of the anterior compartment, the focus has largely remained on the most anterior portion of the native and prosthetic trochlea [8]. However, since patellar loads increase with knee flexion, this region does not represent the most critical segment responsible for patellofemoral joint mechanics, patellar tracking and stability in dynamic conditions [11, 24], which rather corresponds to the trochlear section engaged by the patella between 30° and 70° of the femoral anterior arc [1, 12, 25].

The primary aim of the current study was to define the ‘mid‐flexion trochlear line’ (MTL) and assess its orientation relative to the posterior condylar line. The underlying hypothesis was that the two lines—just as their corresponding axes—are parallel, thereby reinforcing a core principle of kinematic alignment.

## MATERIALS AND METHODS

### Study design and participants

Institutional review board approval (IRB No. 32.19 OSS) was granted for this study and written consent was obtained from all participants. Prospectively collected data of 157 consecutive patients (172 knees) with end stage knee osteoarthritis (Grade IV Kellgren–Lawrence) undergoing calipered KA TKA with the GMK Sphere® (Medacta) between August 2024 and June 2025 at our Institution (Fondazione Policlinico Universitario Campus Bio‐Medico) were obtained. The exclusion criteria were: post‐traumatic osteoarthritis (two patients, two knees), radiographic signs of trochlear dysplasia ≥ type A of the Dejour classification [2] (9 patients, 11 knees), and flexion of the femoral component of >5° on post‐op lateral radiographs (one patient, one knee). After applying these criteria, 145 patients (158 knees) were included in the final analysis.

### Surgical technique and intra‐operative measurements

**Figure 1:**
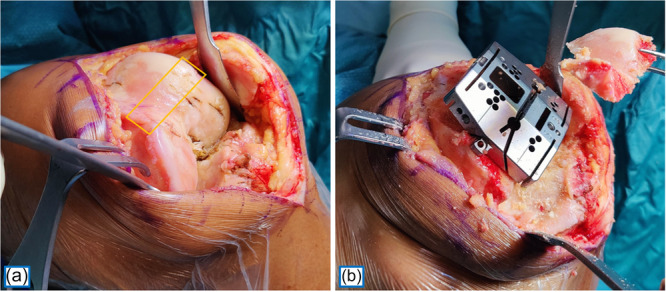
Left knee. Trochlear section corresponding to the anterior chamfer before (a) and after (b) bone resection.

**Figure 2:**
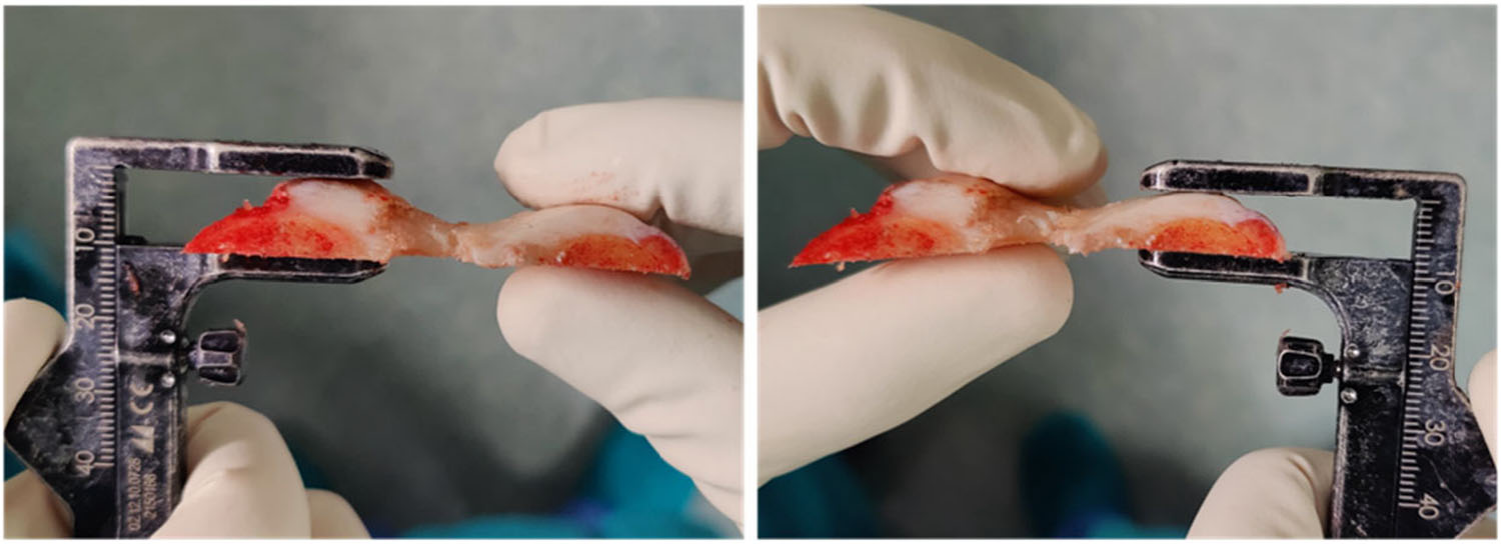
Measurements of the lateral and medial trochlear facets at the anterior chamfer cut, defining the mid‐flexion trochlear line (MTL) orientation.

Following the distal femoral cut, a posterior‐referencing 4‐in‐1 cutting guide was positioned in contact with the posterior condylar line—and thus parallel to the patient's posterior condylar axis—with adjustments made for any cartilage loss, in accordance with the principles of calipered kinematic alignment [9]. After completing the posterior cut, the anterior chamfer cut was performed, and the most prominent points of the lateral and medial trochlear facets—which together define the mid‐flexion trochlear line—were caliper‐measured perpendicular to the cut surface (Figures 1 and 2). Measurements were rounded to the nearest 0.5 mm and adjusted by +1 mm to account for the saw blade thickness, and an additional +2 mm in cases of cartilage wear. All measurements were taken independently and twice—once immediately after the cut and again at the end of the surgery—by two authors (MEC and GG).

The orientation of the MTL relative to the posterior condylar line—referred to as the MTL orientation—was defined by subtracting the medial trochlear facet thickness from the lateral facet thickness. An MTL orientation of 0  ± 1 mm was considered neutral—indicating parallelism with the posterior condylar line—, while positive ( >  +1 mm) and negative values (< –1 mm) indicated a relative internal and external rotation, respectively. Given that the chamfer lies at a 45° angle to the distal condylar line and that femoral components were positioned in a 0°–5° of flexion, the MTL orientation was consistently measured between 45° and 50°, which corresponds to the “functional” section of the trochlea engaged by the patella in mid‐flexion [1, 12].

### Data analysis

Descriptive statistics—including percentage, mean and standard deviation (SD)—were calculated for all variables. The Kolmogorov‐Smirnov test was used to assess the normality of data distribution. To rule out the potential impact of coronal plane parameters on MTL orientation, Pearson's correlation coefficient was used to assess the relationship between MTL orientation and hip‐knee‐ankle angle (HKA), medial proximal tibial angle (MPTA) and lateral distal femoral angle (LDFA), evaluated on full‐limb radiographs [5, 6]. The *t*‐test was performed to compare MTL orientation with the posterior condylar line, and the Two One‐Sided Tests (TOST) was implemented to assess equivalence within a ±1 mm threshold. Measurement reliability was assessed using intra‐class correlation coefficients (ICCs) for intra‐ and inter‐observer agreement.

Statistical significance was set at *p* < 0.05. A post hoc power analysis was conducted to assess the sample size adequacy. All analyses were performed using STATA 18 Software (StataCorp LLC, Lakeway Drive, College Station, Texas, USA).

## RESULTS

The demographic characteristics of the study population are summarised in Table 1. With a sample size of 145 patients (158 knees), the *post hoc* power analysis indicated a power of over 99%.

**Table 1:** Demographic characteristics of the 145 patients (158 prosthesis) included in the study.

Gender (*n*, %)	Male 60 (41.4%)
	Female 85 (58.6%)
Age (years ± SD)	70.4 ± 9.1
BMI (kg/m² ± SD)	27.9 kg/m² ± 5.5
Side (right or left knee)	83 Left
	75 Right
HKA angle (mean ± SD)	−6.4° ± 4.5°
MPTA (mean ± SD)	86.2° ± 2.5°
LDFA (mean ± SD)	89.2° ± 2.8°

Abbreviations: BMI, body mass index; HKA, hip‐knee‐ankle angle; LDFA, lateral distal femoral angle; MPTA, medial proximal tibial angle; SD, standard deviation.

**Figure 3:**
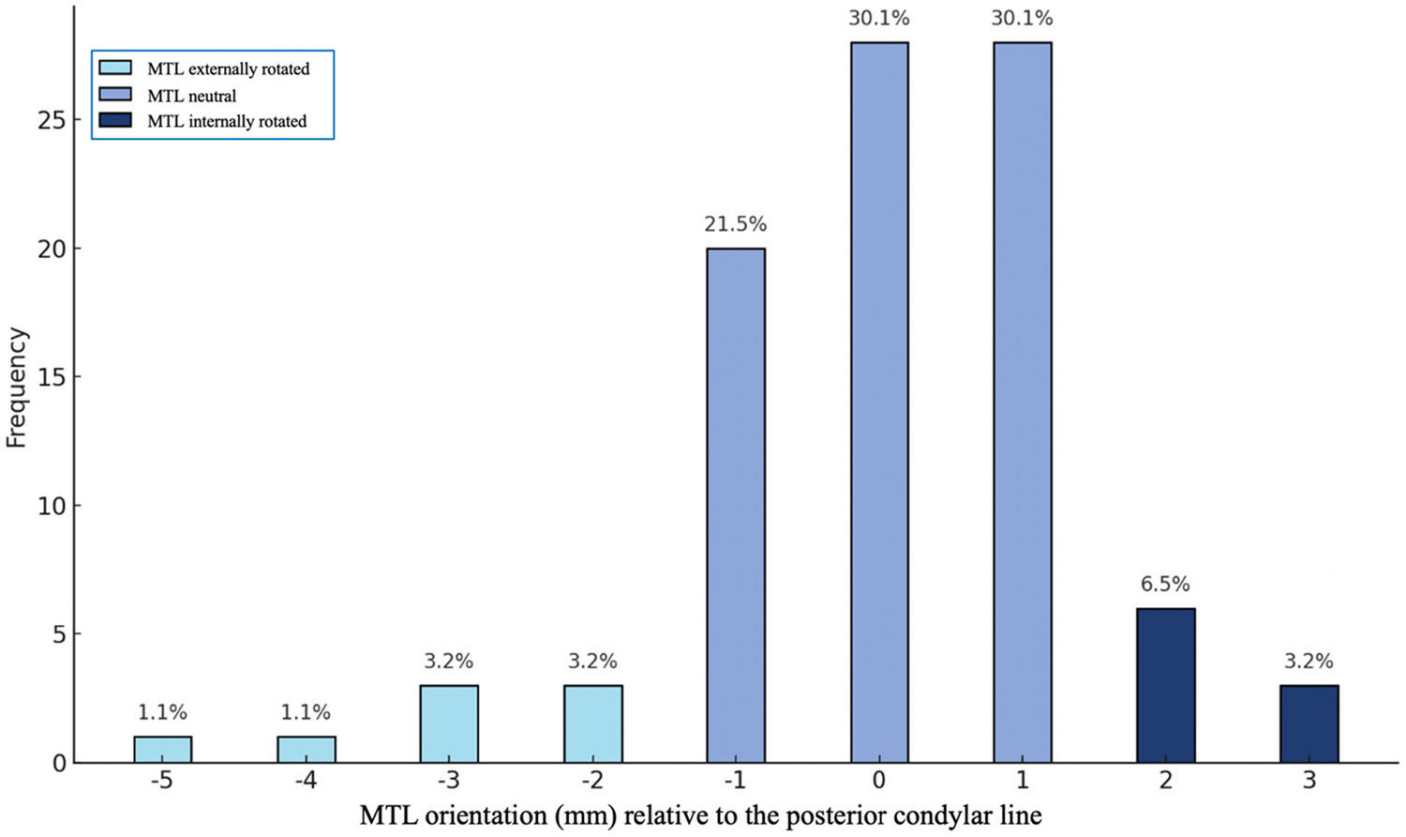
Mid‐flexion trochlear line (MTL) orientation relative to the posterior condylar line expressed in mm.

The mean MTL orientation was 0.1 ± 1.40 mm, with 81.7% of the cases within 0 ± 1 mm, suggesting parallelism between the posterior condylar line and the mid‐flexion trochlear line in the native knee (*p* = 0.709). The maximum positive difference (indicative of internal rotation) was 3 mm and was measured only in 3.2% of cases (Figure 3). Furthermore, statistical equivalence within the ±1 mm threshold was confirmed by the two one‐sided tests (*p* < 0.001). No significant correlation was found between MTL orientation and HKA angle (*R* = 0.236, *p* = 0.090), MPTA (*R* = 0.083, *p* = 0.428) and LDFA (*R* = −0.153, *p* = 0.144).

ICCs for intra‐ and inter‐observer reliability in intraoperative measurements were excellent [7, 16] (0.94 and 0.91, respectively).

## DISCUSSION

The main finding of the current study was that the mid‐flexion trochlear line is parallel to the posterior condylar line in the vast majority of patients, with its orientation being 0 ± 1 mm in 81.7% of the cases. No correlation was found between the MTL orientation and the HKA, MPTA and LDFA, thus confirming the reliability of the posterior condylar line to restore the trochlear orientation irrespective of coronal plane parameters.

Several studies have raised concerns about the potential relative internal rotation of the femoral component and a more medialized trochlear sulcus in kinematic alignment compared to mechanical alignment, due to the absence of the 3° of external rotation typically applied in mechanical alignment [21, 22, 23]. Notably, Klasan et al. [13] compared the impact of three different alignment strategies—MA, KA and FA—on changes in native trochlear orientation. They concluded that KA resulted in the smallest deviation from the native despite a non‐negligible proportion of cases of internal rotation ≥3°. Koh et al. [14] demonstrated that the internal rotation of the femoral component observed in kinematically aligned TKA is associated with a higher incidence of postoperative lateral patellar tilt. Despite that, its clinical impact appears limited, with comparable clinical outcomes and satisfaction scores at both 6 and 24 months postoperatively, relative to patients without tilt. Moreover, the progressive resolution of patellar tilt observed at two years suggests that soft tissue adaptation may compensate over time [14]. Additionally, the overall incidence of patellofemoral complications appears similar between KA and MA techniques, probably because the more valgus‐oriented distal femoral anatomy in KA slackens the lateral retinaculum, thereby reducing the tension often responsible for anterior knee pain [20, 26].

Importantly, previous literature has described femoral rotation referencing to the coordinate system proper of mechanical alignment, thus aiming to achieve parallelism between the trochlear resection plane and the transepicondylar axis [15]. However, the latter does not represent the true kinematic axis around which the patella flexes and extends. The patellar flexion‐extension axis is in fact parallel to posterior condylar axis, as established by the core principles of KA [3, 4, 10, 19]. Our findings demonstrate that referencing the true kinematic axis results in a symmetrical anterior chamfer cut in the vast majority of patients. Interestingly, the maximum internal rotation of the MTL was 3 mm and was measured only in 3.2% of cases. It therefore becomes evident that systematically applying any amount of external rotation in this context would alter the native anatomy of the anterior compartment most of the times, rather than accommodate it. Although image‐based computer assistance could help personalise the anterior chamfer cut to match the native MTL orientation in outlier cases, doing so with current monoblock femoral components would require altering the posterior compartment and flexion gap laxities, which would contradict the resurfacing principle of KA and potentially have deleterious consequences, particularly in cruciate‐retaining implants [17, 18].

Our results differ significantly from those reported by Hess et al. [8]. In their CT‐based study, they found that the ‘anterior trochlear angle’ was parallel to the posterior condylar axis (PCA) in only 14% of cases. This discrepancy arises because the anterior trochlear angle measured on CT by the authors does not correspond to the mid‐flexion trochlear line orientation assessed in our study. Instead, they measured the anterior proximal trochlear surface, which lies proximal to the section where the patella engages and functions as a biomechanical lever. It is this more distal portion—around 45° of the femoral anterior arch [1, 12] —that represents the true functional trochlear line and plays a critical role in patella‐femoral joint kinematics during most activities, both in the native and prosthetic knee, and should be considered and evaluated intraoperatively.

### Limitations

Our study presents few limitations worth mentioning. As a purely descriptive intraoperative study, it lacked both clinical and radiographic follow‐up. While the difference between lateral and medial facets offers a practical and reproducible intraoperative tool to assess axial trochlear orientation, it fails to capture the full three‐dimensional complexity of trochlear morphology, including the medio‐lateral or varus‐valgus orientation of the trochlear groove or its interaction with the extensor mechanism. Future studies assessing the post‐operative variation of these parameters and dynamic analyses—such as fluoroscopy to study patellar tracking in vivo—may provide a more comprehensive understanding of the patellofemoral biomechanics and its clinical implications.

Our results should be interpreted with caution in cases of post‐traumatic knee osteoarthritis following distal femoral fractures, as such injuries may alter the native condylar anatomy. Moreover, patients with radiographic evidence of trochlear dysplasia were excluded from the study and, as a result, our findings may not be generalisable to this subgroup. This exclusion allowed us to focus solely on patients with normal anatomy and to demonstrate that referencing the posterior condylar line can reliably restore normal trochlear anatomy, thus providing a useful framework for correctly managing dysplastic cases as well.

## CONCLUSIONS

Referencing the posterior condylar line accurately restores MTL orientation in the vast majority of patients, irrespective of coronal plane parameters. These findings support the biomechanical rationale of kinematic alignment, dispelling concerns about femoral component internal rotation.

## AUTHOR CONTRIBUTIONS

Stefano Campi and Edoardo Franceschetti were responsible for data collection and conceptualisation. Giancarlo Giurazza was responsible for writing of the manuscript and qualified as corresponding author. Andrea Tanzilli and Biagio Zampogna were responsible for data analysis. Pietro Gregori supervised data acquisition and analysis. Michele Paciotti and Marco Edoardo Cardinale were responsible for realisation of Figures and Tables. Umile Giuseppe Longo and Rocco Papalia were responsible for reviewing and critically revise the manuscript. All authors have given final approval of the version to be published.

## CONFLICT OF INTEREST STATEMENT

The authors declare no conflicts of interest.

## ETHICS STATEMENT

The study was performed in accordance with the ethical standards as laid down in the 1964 Declaration of Helsinki and its later amendments. Institutional review board approval was obtained for this research (IRB no 32.19 OSS). All patients provided legitimate informed consent.

## Data Availability

The data that support the findings of this study are available from the corresponding author, G.G., upon reasonable request.
